# Supplementary material for: ACE2 and TMPRSS2 variants and expression as candidates to sex and country differences in COVID-19 severity in Italy
Source: Aging (Albany NY). 2020 Jun 5;12(11):10087–98. doi: 10.18632/aging.103415 (PMC7346072; doi:10.18632/aging.103415)
Supplement: Supplementary Methods [file aging-12-103415-s001..pdf]

SUPPLEMENTARY MATERIALS

SUPPLEMENTARY METHODS

Sequencing

Whole-exome sequencing (WES) was performed at the Broad Institute (Boston, MA). Demographic characteristics, as well as exome capture methods, sequencing, variant annotation, and data processing of the samples were described previously [1].

Definition of disrupting variants and statistical analysis

Using WES data, we searched the *ACE2* and *TMPRSS2* genes for loss-of-function variants (nonsense, frameshift, splicing, or disrupting missense mutations). Missense variants were considered damaging if they were predicted to be deleterious or possibly deleterious by all the 5 prediction algorithms used: LRT (likelihood ratio test) [2], MutationTaster [3], PolyPhen-2 HumDiv, PolyPhen-2 HumVar [4], and SIFT [5].

The positions of mutations were based on the cDNA reference sequence for *ACE2* and *TMPRSS2* (NM_021804 and NM_005656) with the ATG initiation codon numbered as residue 1 (p.Met1).

Burden test analyses were performed considering only those variants having a minor allele frequency (MAF) <1%. Significance in the differences of MAFs between different populations were calculated using chi-square tests, with the R software (https://www.r-project.org/). A P<0.05 was considered to indicate statistical significance.

Dataset imputation

When missing from exome data, intronic variant frequencies in *TMPRSS2* were retrieved from SNP-array data obtained from the same Italian cohort. Genome-wide genotyping was performed at the Broad Institute. Genotyping details and data processing of the samples have been already described [6].

Imputation was performed remotely using the Michigan Imputation Server (https://imputationserver.sph.umich.edu) [7], using the 1000G Phase 3 v5 as reference panel, ShapeIT v2.r790 for the phasing step [8], and Minimac3 [7] as imputation software. The imputed dataset was then filtered to retain only those variants with r^2>0.3.

Datasets and statistical power estimations

For expression data analyses, we took advantage of microarray data reported in the GEO repository (https://www.ncbi.nlm.nih.gov/geo/). We specifically searched for the wider datasets reporting expression data on normal lung tissues derived from individuals whose sex and geographical origin were specified (search done by keywords, filters based on the number of available samples in the dataset, and by a final manual inspection of the retrieved data). This search allowed the identification of two datasets: GSE66499 and GSE19804, for a total of 115 samples from male individuals, and 135 samples from female subjects. Indeed, it is difficult to provide an accurate power estimate for a microarray study. Among others, [9] suggested that a sample size of 20 is necessary, at a P value of 0.01 and 90% power, to detect a two-fold change in the 75% least variable genes in a microarray study. Based on this observation, the data available through the GSE66499 and GSE19804 datasets were considered reasonably powered to identify possible altered levels in the ACE2 and TMPRSS2 genes.

As for genotype data, from one side we took advantage of exome and SNP-array in-house data on ~3,500 individuals; [1, 6], from the other of exome and genome data on the largest dataset freely accessible online, i.e. the GnomAD repository (https://gnomad.broadinstitute.org/). For GnomAD data, we extracted allele/genotype frequencies available for East Asian and European individuals, for a total of at least 9,967 and 64,302 subjects, respectively. The use of such large cohorts ensured us to be sufficiently powered to detect significant differences in allele frequencies between the analyzed populations. As an example, a sample size of 2,000 pairs has an approximately 80% power of detecting a significant allele difference at P<0.05 if the frequency of the rare allele is 2%. For higher frequencies of 10% or more, the power of detection increases to more than 90%.

REFERENCES

1. Do R, Stitziel NO, Won HH, Jørgensen AB, Duga S, Angelica Merlini P, Kiezun A, Farrall M, Goel A, Zuk O, Guella I, Asselta R, Lange LA, et al, and NHLBI Exome Sequencing Project. Exome sequencing identifies rare LDLR and APOA5 alleles conferring risk for myocardial infarction. Nature. 2015; 518:102–6. https://doi.org/10.1038/nature13917 PMID:25487149

2. Chun S, Fay JC. Identification of deleterious mutations within three human genomes. Genome Res. 2009; 19:1553–61. https://doi.org/10.1101/gr.092619.109 PMID:19602639

3. Schwarz JM, Rödelsperger C, Schuelke M, Seelow D. MutationTaster evaluates disease-causing potential of sequence alterations. Nat Methods. 2010; 7:575–6. https://doi.org/10.1038/nmeth0810-575 PMID:20676075

4. Adzhubei IA, Schmidt S, Peshkin L, Ramensky VE, Gerasimova A, Bork P, Kondrashov AS, Sunyaev SR. A method and server for predicting damaging missense mutations. Nat Methods. 2010; 7:248–9. https://doi.org/10.1038/nmeth0410-248 PMID:20354512

5. Kumar P, Henikoff S, Ng PC. Predicting the effects of coding non-synonymous variants on protein function using the SIFT algorithm. Nat Protoc. 2009; 4:1073–81. https://doi.org/10.1038/nprot.2009.86 PMID:19561590

6. Kathiresan S, Voight BF, Purcell S, Musunuru K, Ardissino D, Mannucci PM, Anand S, Engert JC, Samani NJ, Schunkert H, Erdmann J, Reilly MP, Rader DJ, et al, Myocardial Infarction Genetics Consortium, and Wellcome Trust Case Control Consortium. Genome-wide association of early-onset myocardial infarction with single nucleotide polymorphisms and copy number variants. Nat Genet. 2009; 41:334–41. https://doi.org/10.1038/ng.327 PMID:19198609

7. Das S, Forer L, Schönherr S, Sidore C, Locke AE, Kwong A, Vrieze SI, Chew EY, Levy S, McGue M, Schlessinger D, Stambolian D, Loh PR, et al. Next-generation genotype imputation service and methods. Nat Genet. 2016; 48:1284–87. https://doi.org/10.1038/ng.3656 PMID:27571263

8. Delaneau O, Coulonges C, Zagury JF. shape-IT: new rapid and accurate algorithm for haplotype inference. BMC Bioinformatics. 2008; 9:540. https://doi.org/10.1186/1471-2105-9-540 PMID:19087329

9. Wei C, Li J, Bumgarner RE. Sample size for detecting differentially expressed genes in microarray experiments. BMC Genomics. 2004; 5:87. https://doi.org/10.1186/1471-2164-5-87 PMID:15533245
